# Serum ammonia is a strong prognostic factor for patients with acute-on-chronic liver failure

**DOI:** 10.1038/s41598-020-73603-1

**Published:** 2020-10-12

**Authors:** Chenxia Hu, Kaizhou Huang, Lingfei Zhao, Fen Zhang, Zhongwen Wu, Lanjuan Li

**Affiliations:** 1 Collaborative Innovation Center for the Diagnosis and Treatment of Infectious Diseases, State Key Laboratory for the Diagnosis and Treatment of Infectious Diseases, First Affiliated Hospital, College of Medicine, Zhejiang University, Hangzhou, Zhejiang People’s Republic of China; 2 National Clinical Research Center for Infectious Diseases, The First Affiliated Hospital, School of Medicine, Zhejiang University, Hangzhou, Zhejiang People’s Republic of China; 3 Shantou Central Hospital, Affiliated Shantou Hospital of Sun Yat-Sen University, Shantou, 515041 Guangdong People’s Republic of China; 4 Key Laboratory of Kidney Disease Prevention and Control Technology, Kidney Disease Center, Institute of Nephrology, First Affiliated Hospital, College of Medicine, Zhejiang University, Hangzhou, Zhejiang People’s Republic of China

**Keywords:** Gastroenterology, Hepatology, Liver diseases

## Abstract

Ammonia is thought to be central to the pathogenesis of hepatic encephalopathy (HE), but its prognostic role in acute-on-chronic liver failure (ACLF) is still unknown. We aimed to determine the association between serum ammonia level and short-term prognosis in ACLF. Furthermore, we performed an in-depth evaluation of the independent effect of serum ammonia level on the short-term prognosis of hepatitis B virus (HBV) reactivation-induced ACLF patients. We identified 174 patients as part of prospective observational studies in patients with ACLF. Plasma ammonia levels were measured on admission, and several prognostic scores were used to determine the prognostic effect of ammonia. The 28-day patient survival was determined. Receiver operating characteristic analysis was used to identify the cut-off points for ammonia values, and multivariable analysis was performed using the Cox proportional hazard regression model. Plasma ammonia was significantly higher in nonsurvivors (83.53 ± 43.78 versus 67.13 ± 41.77 µmol/L, P = 0.013), and ACLF patients with hyperammonemia had significantly higher 28-day mortality than those without hyperammonemia. Ammonia was also closely related to ACLF grade (P < 0.001) and organ failure, including liver (P = 0.048), coagulation (P < 0.001) and brain (P < 0.001). HBV reactivation serves as the main precipitating factor in the ACLF population. Subgroup analysis showed that ammonia is also a strong prognostic factor in the HBV reactivation-induced ACLF population. Ammonia level is closely correlated with failure of other organs and is an independent risk factor for mortality in ACLF and the special population defined as HBV reactivation-related ACLF. Based on the results from our study, we measured serum ammonia in the population with ACLF, which strongly indicates their prognosis. It serves as an important biomarker and a therapeutic target.

## Introduction

Multiple studies have highlighted that hyperammonemia plays a critical role in the development of hepatic encephalopathy (HE) in patients with liver cirrhosis and other liver diseases. A large amount of serum ammonia escapes liver metabolism in acute liver failure (ALF) patients, and high ammonia concentrations are closely related to a high incidence of cerebral edema and herniation^1^. Hyperammonemia may aggravate liver injury by impairing liver cells, accelerating immune dysfunction, activating hepatic stellate cell proliferation and reducing liver recovery^2^. Although ammonia is not closely related to HE grade/coma score in patients with liver cirrhosis, those patients with Grade 4 HE have higher infection and systemic inflammation in vivo as they had a higher systemic inflammation score and SOFA score^3^. Ammonia is highly related to the risk and frequency of HE episodes, while glycerol phenylbutyrate can decrease the level of ammonia and risk of HE episodes regardless of the basal level of ammonia^4^. Regardless of ACLF severity, patients with HE had higher mortality. Moreover, they demonstrated that ammonia, abnormal cerebral oxygen consumption and systemic inflammation may play critical roles in HE episodes^5^. In a recent study, Shalimar et al. included 498 patients with liver cirrhosis and showed that the serum ammonia level is highly correlated with the severity of HE and the incidence of 28 deaths in patients with liver cirrhosis^2^. In patients with acutely decompensated cirrhosis, an admission ammonia level > 60 µmol/L had a higher 90-day and 30-day risk of death or transplantation and a lower 90-day transplant-free survival rate^6^. The progression of acute-on-chronic liver failure (ACLF) based on chronic liver diseases is hard to control since these patients are characterized by precipitating events, acute deterioration, and severe hepatic abnormalities^7^. The Asian Pacific Association for the Study of the Liver (APASL) defined ACLF as “an acute hepatic insult manifesting as jaundice and coagulopathy, complicated within 4 weeks by ascites and/or HE in 2019”^8^. Patients with chronic hepatitis B virus (HBV) infection easily progress into ACLF since chronic liver diseases rapidly deteriorate, accompanied by multiorgan failure and high short-term mortality^7^. Current studies clarified that approximately 40% to 60% of ACLF cases occur in patients with flare-up of chronic HBV and that hepatitis B flare-up is commonly spontaneous according to further analysis^9^. HBV reactivation always occurs in patients with inappropriate withdrawal or usage of nucleoside analogs, resistance to nucleoside analogs, immunosuppressors, chemotherapy and other reasons^10^. According to current studies, the relationship between serum ammonia levels and patients with ACLF is still undetermined, especially in those populations with HBV reactivation-induced ACLF.

In this study, we aimed to determine the association between serum ammonia level and short-term prognosis in ACLF. Furthermore, we performed an in-depth evaluation of the independent effect of serum ammonia level on the short-term prognosis of HBV reactivation-induced ACLF patients.

## Methods

### Patients

**Figure 1:**
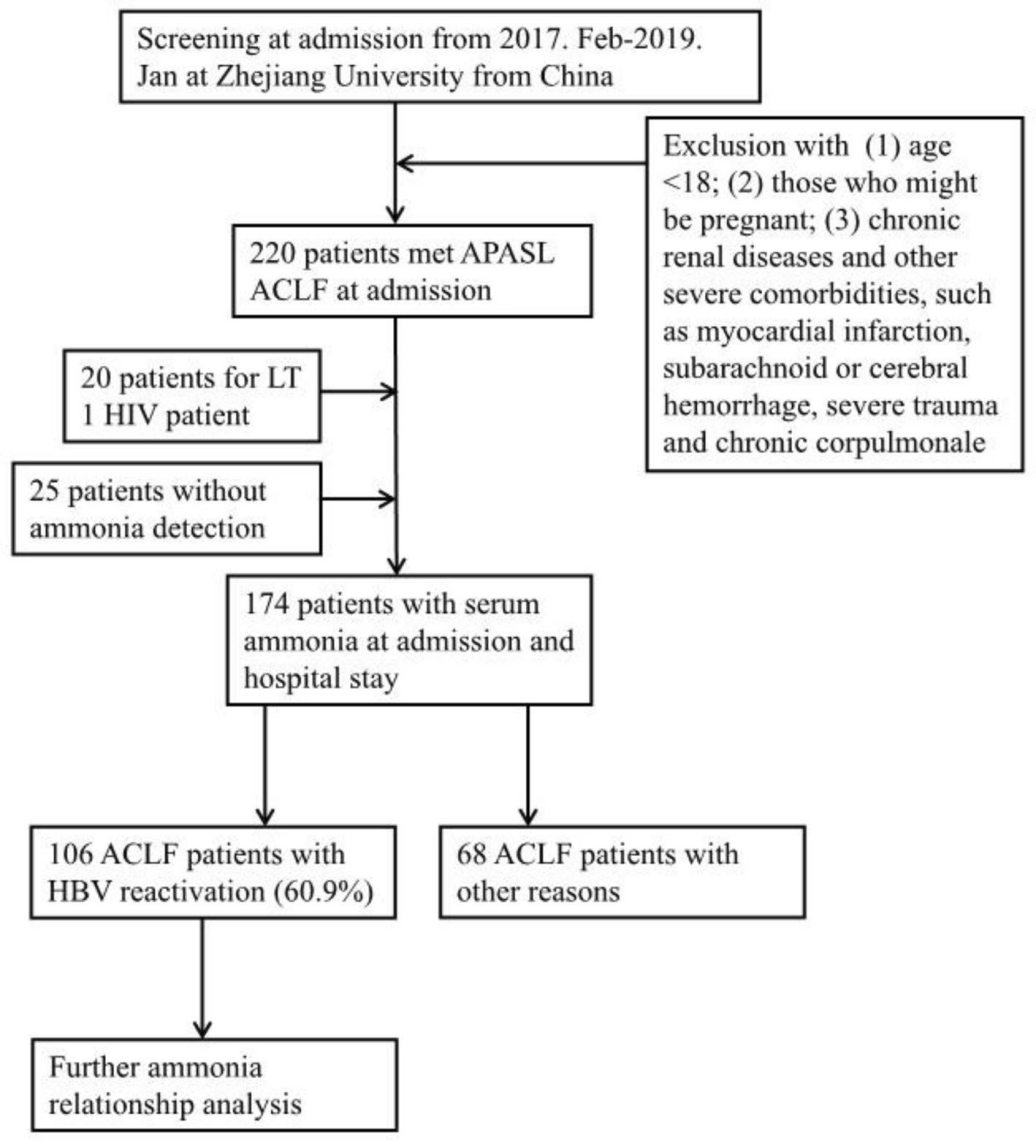
Flow chart of ACLF patient admission.

All methods were carried out in accordance with relevant guidelines and regulations. Written informed consent was obtained from all patients or their legal surrogates prior to enrollment. All patients were screened according to the APASL ACLF definition in 2019 at admission, and they were subsequently hospitalized and treated at the Department of Infectious Disease Department of the First Affiliated Hospital of Zhejiang University from February 2017 to January 2020 (Fig. 1). The exclusion criteria included (1) age < 18; (2) pregnancy; (3) chronic renal diseases and other severe comorbidities, such as myocardial infarction, subarachnoid or cerebral hemorrhage, severe trauma and chronic corpulmonale; (4) human immunodeficiency virus (HIV) infection; (5) acceptance of liver transplantation; and (5) no detection of serum ammonia. HBV reactivation was defined as a ≥ 2 log increase in the HBV DNA level from a previously stable baseline level or a level ≥ 100 IU/mL in patients whose HBV DNA had been undetectable or ≥ 20,000 IU/mL in those negative for HBV DNA at baseline^11,12^. We used CLIF criteria for further classification^13^. Briefly, liver failure (TBil ≥ 12 mg/dL), brain failure (grade III–IV HE), kidney failure (serum creatinine ≥ 2 mg/dL), coagulation failure (INR ≥ 2.5 or platelet count ≤ 20 × 109/L), circulation failure (mean arterial pressure < 70 mmHg or treatment with a vasoactive agent), and lung failure (PaO2/FiO2 ≤ 200 or SpO2/FiO2 ≤ 214) were recorded. This study was approved by the Clinical Research Ethics Committees of the First Affiliated Hospital, Zhejiang University School of Medicine. After exclusion, a total of 174 patients were recruited, and their baseline venous ammonia was measured at admission. We started the follow-up of all included patients at the onset of hospital admission and obtained all prognostic information from medical records or telephone contact after they were discharged. The primary endpoint of the study was 28-day mortality, and we recorded their survival time up to 28 days.

### Measurement of parameters/data collection

We collected the following clinical and demographic information in a prespecified datasheet: age; sex; etiologies of cirrhosis; precipitating factors; complications and laboratory parameters; organ failure events; ACLF grade and prognosis. Acute precipitating factors were categorized into HBV reactivation, hepatotoxic drugs, hepatitis A virus (HAV) or hepatitis E virus (HEV) superimposed infection, alcohol drinking, surgery, and unknown cause. Liver cirrhosis was diagnosed based on symptoms and signs of portal hypertension and findings on ultrasonography, computed tomography or magnetic resonance imaging. Ascites was confirmed via paracentesis, abdominal imaging and other clinical evidence. HE assessment and grading employed the West Haven criteria^14^. Gastrointestinal hemorrhage was diagnosed by a positive fecal occult blood test or the presence of blood in vomit. Spontaneous bacterial peritonitis was explored via ascites examination or laboratory culture. Infection was diagnosed as follows: (1) spontaneous bacterial peritonitis, polymorphonuclear cell count in ascitic fluid > 250/mL; (2) pneumonia, new pulmonary infiltrate in radiologic imaging plus the presence of any respiratory symptoms (cough, sputum, dyspnea, or pleuritic pain), any findings on auscultation (rales or crepitation), core body temperature > 38 °C or < 36 °C, and WBC count > 10,000/mm^3^ or < 4000/mm^3^; (3) urinary tract infection, WBC count in urine > 10/high power field with positive urine culture and urinary irritation symptoms; and (4) other bacterial infections, including catheter-related infection, osteoarticular infection, skin infection, and bacteremia of unknown cause^15^. As previous studies highlighted that there was no significant difference between venous and arterial ammonia values with respect to HE^16^, we collected venous ammonia for data analysis. ACLF grade was further identified based on the European Association for the Study of the Liver-Chronic Liver Failure (EASL-CLIF) criteria as follows: Grade 0: without organ function failure; nonkidney single organ function failure manifesting as a serum creatinine level of < 1.5 mg/dL without HE; noncerebral single organ function failure manifesting as a serum creatinine level < 1.5 mg/dL; Grade 1: single kidney function failure; single organ function failure either of liver, blood coagulation, circulatory or respiratory function manifesting as a serum creatinine level ≥ 1.5 mg/dL but < 2 mg/dL and/or grade 1 or grade 2 HE; single cerebral function failure manifesting as a serum creatinine level ≥ 1.5 mg/dL but < 2 mg/dL; Grade 2: double organ function failures; Grade 3: triple organ function failures^13^.

### ACLF treatments

The ACLF patients were managed according to established guidelines^8,17,18^. To eliminate or control precipitating factors/complications, patients who were HBV DNA positive were immediately given nucleoside analogs daily according to their previous usage of nucleoside analogs. Patients using hepatotoxic drugs or actively drinking alcohol were required to stop using or abstain from alcohol. Patients with bacterial infection were immediately treated with empirical antibiotic therapy, and adjustment of antibiotic therapy was based on bacterial culture and antibiotic sensitivity tests. In addition, weight-based intravenous albumin was used, especially in patients with spontaneous bacterial peritonitis (SBP); furthermore, all patients with acute variceal bleeding received IV somatostatin, proton pump inhibitors, and antibiotic prophylaxis. For those with uncontrolled hemorrhage resulting from pharmacological therapy, Sengstaken-Blackmore tube or urgent therapeutic endoscopy were performed. Diagnostic abdominocentesis was performed in patients with ascites to examine whether SBP was present. Those with moderate ascites were treated with restriction of sodium intake and/or diuretics. Paracentesis combined with IV albumin was used in those with large or refractory ascites. Patients with renal failure were treated with IV albumin, vasoconstrictors and even renal replacement therapy. Patients with HE were given l-ornithine aspartate, lactulose, antibiotics, and discontinuation of potential precipitating events. Fluid replacement was performed in patients with mean arterial pressure (MAP) < 70 mmHg, and vasoactive agents were used when necessary. Oxygen therapy was performed in patients with decreased PaO_2_ or SpO_2_. The choice of nasal catheter, mask, or venturi mask oxygen inhalation or mechanical ventilation was based on the severity of respiratory dysfunction. Nutritional support is also the basis of all patients.

### Statistical analysis

Prognostic models used in predicting the 28-day mortality of ACLF patients included the Model for End-Stage Liver Disease (MELD); MELD sodium (MELD-Na); the integrated MELD (iMELD); CLIF Consortium Organ Failure score (CLIF-C OFs); and CLIF-Consortium-ACLF (CLIF-C-ACLF) score. The MELD score (range 6–40) was calculated as follows: 9.6*loge [creatinine (mg/dL)] + 3.8*loge [bilirubin (mg/dl)] + 11.2*loge (INR) + 6.43* (etiology: 0 if cholestatic or alcoholic, 1 otherwise)^19^. MELD-Na = MELD + 1.59 (135 − Na)^20^. The iMELD model for ACLF patients was recently proposed and calculated as follows: MELD + 0.3*Age [years] − 0.7*Na [mmol/L] + 100^21^. The CLIF-C-ACLF score was calculated as follows: 10*[0.33*CLIF-C OFs + 0.04*Age + 0.63*Ln (white-cell count) – 2]^22^. Organ failures were determined according to the CLIF Consortium Organ Failure score (CLIF-C OFs)^22^.

Continuous variables that were normally distributed were expressed as the mean ± standard deviation, and other variables were expressed as the median with interquartile ranges. Categorical data are presented as proportions. Comparison of demographics and clinical features was performed using the chi-square or Fisher’s exact test for categorical variables. Continuous data between two groups were compared by Student’s t test or Mann–Whitney’s U test. Candidate variables (P < 0.10) after a bivariate analysis were entered into multivariable analysis by the Cox proportional hazard regression model^2^. Receiver operating characteristic curves were used to identify the cut-off points for baseline ammonia values. The Kaplan–Meier method was used to generate survival curves. The data were analyzed using SPSS statistics software (version 19.0; SPSS, Chicago, IL) and GraphPad software (version 5.0.1; MedCalc Software, Ostend, Belgium).

## Results

### Patients' baseline characteristics

Baseline demographic characteristics are shown in Table 1. Overall mortality at 28 days was 41.4% (n = 72). Nonsurvivors were older (55.74 ± 12.51 versus 51.61 ± 10.30, P = 0.018). The most common precipitating event of ACLF was HBV reactivation (52.8% in nonsurvivors and 67.6% in survivors), and the overall rate of HBV reactivation in the ACLF population was 61.5% (n = 107). Nonsurvivors had a higher frequency of severe ACLF and organ failures, including liver, brain, and coagulation (P < 0.05). The complications did not differ between the survivors and nonsurvivors. Ammonia, total bilirubin (TB), direct bilirubin (DB), and prothrombin time (PT) levels were higher in nonsurvivors than in survivors (83.53 ± 43.78 µmol/L versus 67.13 ± 41.77 µmol/L, P = 0.013; 23.42 ± 8.17 versus 18.19 ± 8.66, P < 0.001; 17.11 ± 6.04 versus 12.90 ± 6.32, P < 0.001; 27.15 ± 9.10 versus 22.93 ± 4.49, P < 0.001), while thyroid stimulating hormone (TSH) and lymphocyte levels were lower in nonsurvivors than in survivors (0.68 ± 1.22 versus 1.07 ± 1.00, P = 0.023; 0.90 ± 0.59 versus 1.25 ± 1.01, P = 0.009). Baseline MELD, MELD-Na, iMELD, CLIF-OF and CLIF-C ACLF scores were all higher in nonsurvivors than in survivors (P ≤ 0.001).

**Table 1:** Comparison of demographic and clinical characteristics of patients at diagnosis of ACLF.

Baseline characteristics	Survivors (N = 102)	Nonsurvivors (N = 72)	P
**Predisposition**			
Age	51.61 ± 10.30	55.74 ± 12.51	0.018
Male sex	72 (70.6%)	48 (66.7%)	0.582
**Etiology, N (%)**			
HBV	88 (86.3%)	57 (79.2%)	0.215
Autoimmune	1 (1%)	1 (1.4%)	1
PBC	3 (2.9%)	3 (4.2%)	0.693
HCV	1 (1%)	1 (1.4%)	1
Alcohol	31 (30.4%)	30 (41.7%)	0.125
HBV + alcohol	21 (20.6%)	20 (27.8%)	0.271
Wilson disease	0 (0%)	1 (1.4%)	0.414
Schistosomes	1 (1%)	5 (6.9%)	0.083
Others	2 (2%)	1 (1.4%)	1
**Precipitating event, N (%)**			
HBV reactivation	69 (67.6%)	38 (52.8%)	0.047
Drug use	10 (9.8%)	12 (16.7%)	0.18
HAV or HEV	1 (1%)	0 (0%)	1
Alcoholism	4 (3.9%)	2 (2.8%)	1
Surgery	0 (0%)	0 (0%)	N/A
Others	16 (15.7%)	20 (27.8%)	0.052
**Complications, N (%)**			
SBP	22 (21.6%)	13 (18.1%)	0.569
Gastrointestinal hemorrhage	9 (8.8%)	11 (15.3%)	0.189
Ascites	99 (97.1%)	71 (98.6%)	0.643
Infection	10 (9.8%)	14 (19.4%)	0.069
HE	41 (40.2%)	30 (41.7%)	0.846
**Organ failure, N (%)**			
Liver	72 (70.6%)	64 (88.9%)	0.004
Kidney	2 (2%)	3 (4.2%)	0.65
Cerebral	6 (5.9%)	21 (29.2%)	< 0.001
Coagulation	18 (17.6%)	24 (33.3%)	0.017
Circulation	6 (5.9%)	11 (15.3%)	0.04
Lung	11 (10.8%)	7 (9.7%)	0.821
**ACLF grade**			
ACLF grade 0	76 (74.5%)	31 (43.1%)	< 0.001
ACLF grade 1	0 (0%)	2 (2.8%)	
ACLF grade 2	19 (18.6%)	19 (26.4%)	
ACLF grade 3	7 (6.9%)	20 (27.8%)	
**Laboratory parameters**			
Ammonia (μmol/L)	67.13 ± 41.77	83.53 ± 43.78	0.013
TSH (mIU/L)	1.07 ± 1.00	0.68 ± 1.22	0.023
Lymphocyte (10e9/L)	1.25 ± 1.01	0.90 ± 0.59	0.009
TB (mg/dL)	18.19 ± 8.66	23.42 ± 8.17	< 0.001
DB (mg/dL)	12.90 ± 6.32	17.11 ± 6.04	< 0.001
PT (s)	22.93 ± 4.49	27.15 ± 9.10	< 0.001
**Prognostic score**			
MELD	22.22 ± 5.70	26.45 ± 7.36	< 0.001
MELD-Na	20.01 ± 9.83	25.52 ± 11.25	0.001
iMELD	42.23 ± 7.43	48.26 ± 9.76	< 0.001
CLIF-C OF	8.24 ± 1.78	9.76 ± 2.11	< 0.001
CLIF-C ACLF	39.59 ± 8.06	47.05 ± 10.29	< 0.001

### Comparison of demographic and clinical characteristics in ACLF patients with and without elevated ammonia

Overall, 58 patients (33.3%) had baseline ammonia ≥ 89 µmol/L (Table 2). In the group with ammonia ≥ 89 µmol/L, the incidence of overt HE did not differ from patients with ammonia ≤ 89 µmol/L (P = 0.081). An ammonia level ≥ 89 µmol/L was associated with a higher frequency of organ failure (cerebral 36.2% versus 5.2% (P < 0.001); coagulation 39.7% versus 16.4% (P < 0.001)). In addition, an ammonia level ≥ 89 µmol/L was associated with severe ACLF in our study (P < 0.001). In the group with ammonia ≥ 89 µmol/L, alkaline phosphatase (ALP) and PT levels were higher than those with ammonia ≤ 89 µmol/L (172.43 ± 105.49 versus 136.97 ± 54.55, P = 0.019; 26.28 ± 6.52 versus 23.88 ± 7.23, P = 0.035), while triglyceride (TG) and very low density lipoprotein (VLDL) levels were lower than those with ammonia ≤ 89 µmol/L (1.17 ± 0.45 versus 1.34 ± 0.57, P = 0.049; 0.86 ± 0.57 versus 1.07 ± 0.66, P = 0.04). The prognostic scores, including CLIF-C OF and CLIF-C ACLF, were higher in patients with ammonia ≥ 89 µmol/L than in those with ammonia ≤ 89 µmol/L (9.88 ± 2.24 versus 8.36 ± 1.76, P < 0.001; 47.04 ± 10.57 versus 40.49 ± 8.55, P < 0.001).

**Table 2:** Comparison of demographic and clinical characteristics in ACLF patients with and without elevated ammonia.

	Ammonia < 89 (n = 116)	Ammonia ≥ 89 (n = 58)	P
Male sex	82 70.7	38 65.5	0.487
**Complications, N (%)**			
SBP	27 (23.3%)	8 (13.8%)	0.141
Gastrointestinal hemorrhage	11 (9.5%)	9 (15.5%)	0.239
Ascites	112 (96.6%)	58 (100%)	0.303
Infection	12 (10.3%)	12 (20.7%)	0.062
HE	49 (36.2%)	29 (50%)	0.081
**Laboratory parameters**			
ALP (U/L)	136.97 ± 54.55	172.43 ± 105.49	0.019
Triglyceride (mmol/L)	1.34 ± 0.57	1.17 ± 0.45	0.049
VLDL (mmol/L)	1.07 ± 0.66	0.86 ± 0.57	0.04
PT (s)	23.88 ± 7.23	26.28 ± 6.52	0.035
**Organ failures**			
Liver	86 (74.1%)	50 (86.2%)	0.069
Kidney	3 (2.6%)	2 (3.4%)	1
Cerebral	6 (5.2%)	21 (36.2%)	< 0.001
Coagulation	19 (16.4%)	23 (39.7%)	0.001
Circulation	8 (6.9%)	9 (15.5%)	0.071
Lung	10 (8.6%)	8 (13.8%)	0.291
**ACLF grade**			
ACLF grade 0	85 (73.3%)	22 (37.9%)	< 0.001
ACLF grade 1	2 (1.7%)	0 (0%)	
ACLF grade 2	20 (17.2%)	18 (31%)	
ACLF grade 3	9 (7.8%)	18 (31%)	
28-day mortality	38 (32.8%)	34 (58.6%)	0.001
Survival time	23.12 ± 8.07	20.07 ± 8.88	0.024
**Prognostic score**			
CLIF-C OF	8.36 ± 1.76	9.88 ± 2.24	< 0.001
CLIF-C ACLF	40.49 ± 8.55	47.04 ± 10.57	< 0.001

### Associations of clinical parameters and prognostic scoring systems with serum ammonia in ACLF patients

The correlations between baseline serum ammonia level and laboratory data, organ failure and prognostic scores were explored (Table 3). Serum ammonia levels were inversely correlated with TG levels (r =  − 0.156, P = 0.04) and VLDL levels (r =  − 0.197, P = 0.009). The serum ammonia level was positively correlated with the PT level (r = 0.216, P = 0.004). The serum ammonia level was positively correlated with organ failure (liver, r = 0.129, P = 0.04; cerebral, r = 0.338, P < 0.001; coagulation, r = 0.241, P < 0.001) and ACLF grade (r = 0.342, P < 0.001). The serum ammonia level was positively correlated with prognostic predictive scores (CLIF-OF: r = 0.382, P < 0.001; CLIF-C ACLFs: r = 0.369, P < 0.001).

**Table 3:** Associations of clinical parameters and prognostic scoring systems with serum ammonia.

Variable	Regression coefficient	95% CI	P
**Laboratory parameter**			
TG (mmol/L)	− 0.156	− 0.298 to − 0.014	0.04
VLDL (mmol/L)	− 0.197	− 0.320 to − 0.053	0.009
PT (s)	0.216	0.101 to 0.372	0.004
**Organ failure**			
Liver	0.129	0.004 to 0.240	0.04
Kidney	0.003	− 0.119 to 0.106	0.957
Cerebral	0.338	0.239 to 0.426	< 0.001
Coagulation	0.241	0.128 to 0.342	< 0.001
Circulation	0.036	− 0.104 to 0.172	0.567
Lung	0.041	− 0.094 to 0.169	0.509
ACLF grade	0.342	0.21 to 0.486	< 0.001
**Prognostic score**			
MELD	0.134	0.015 to 0.261	0.078
MELD-Na	0.063	− 0.062 to 0.189	0.410
iMELD	0.135	0.011 to 0.261	0.076
CLIF-C OF	0.382	0.264 to 0.504	< 0.001
CLIF-C ACLF	0.369	0.246 to 0.49	< 0.001
28-day mortality	0.159	0.283 to 0.209	0.011

**Figure 2:**
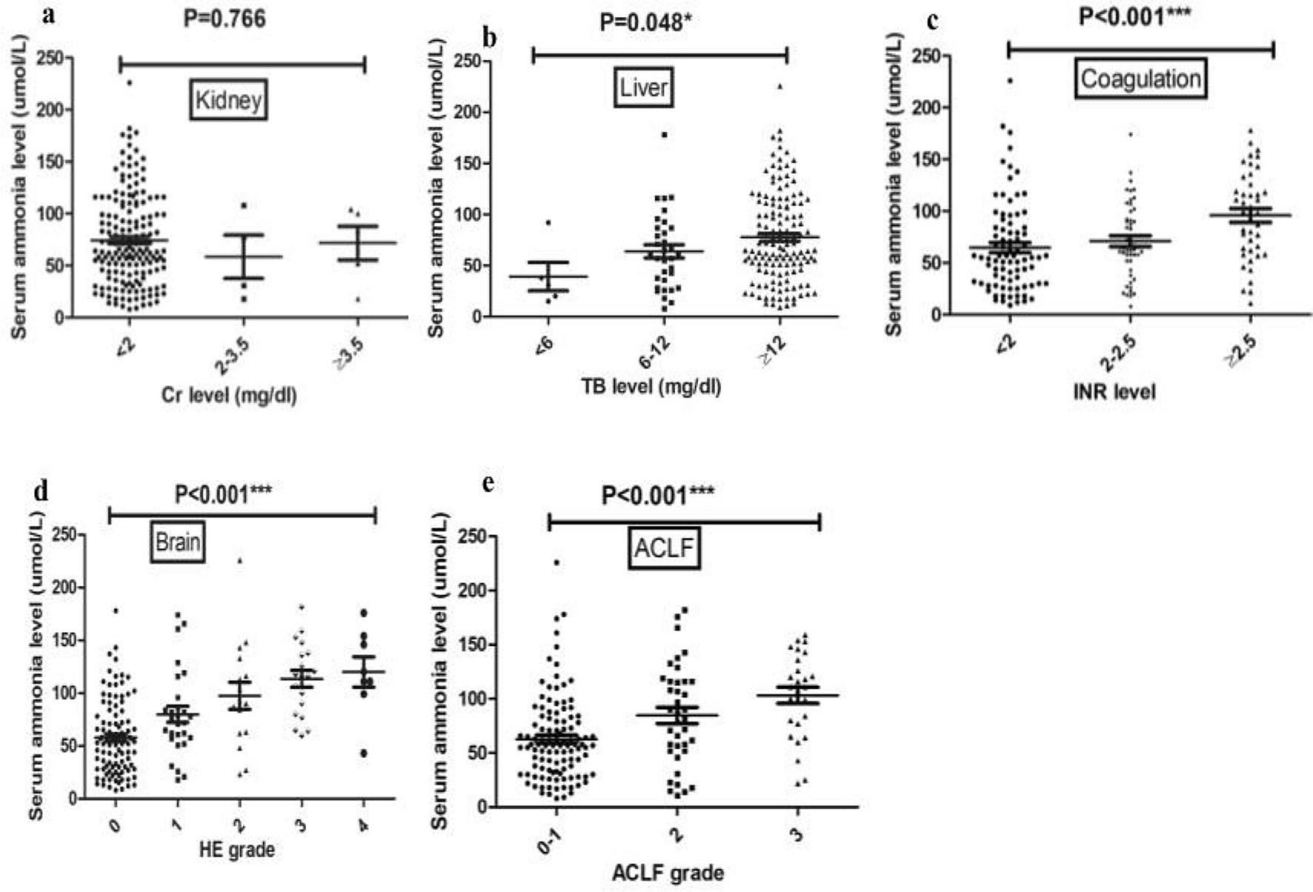
Ammonia levels and organ injury. Comparisons of ammonia levels among subjects within subgroups of ACLF subjects according to CLIF-OF score and EASL-CLIF: (**a**) liver; (**b**) kidney; (**c**) coagulation; (**d**) brain; (**e**) ACLF grade.

Serum ammonia levels at admission were further compared among subjects with various degrees of organ injury according to the CLIF-OF scoring system (Fig. 2). Serum ammonia levels were comparable in subjects with different levels of Cr (P = 0.766) (Fig. 2a), while they were higher in subjects with liver failure (P = 0.048) (Fig. 2b), more severe coagulation and brain injuries (P < 0.001) (Fig. 2c,d). Moreover, serum ammonia levels were higher in subjects with ACLF grade 2 and grade 3 (P < 0.001) (Fig. 2e).

### Receiver operating characteristic (ROC) curve analysis and comparisons in the ACLF and HBV reactivation-induced ACLF subgroups

**Figure. 3:**
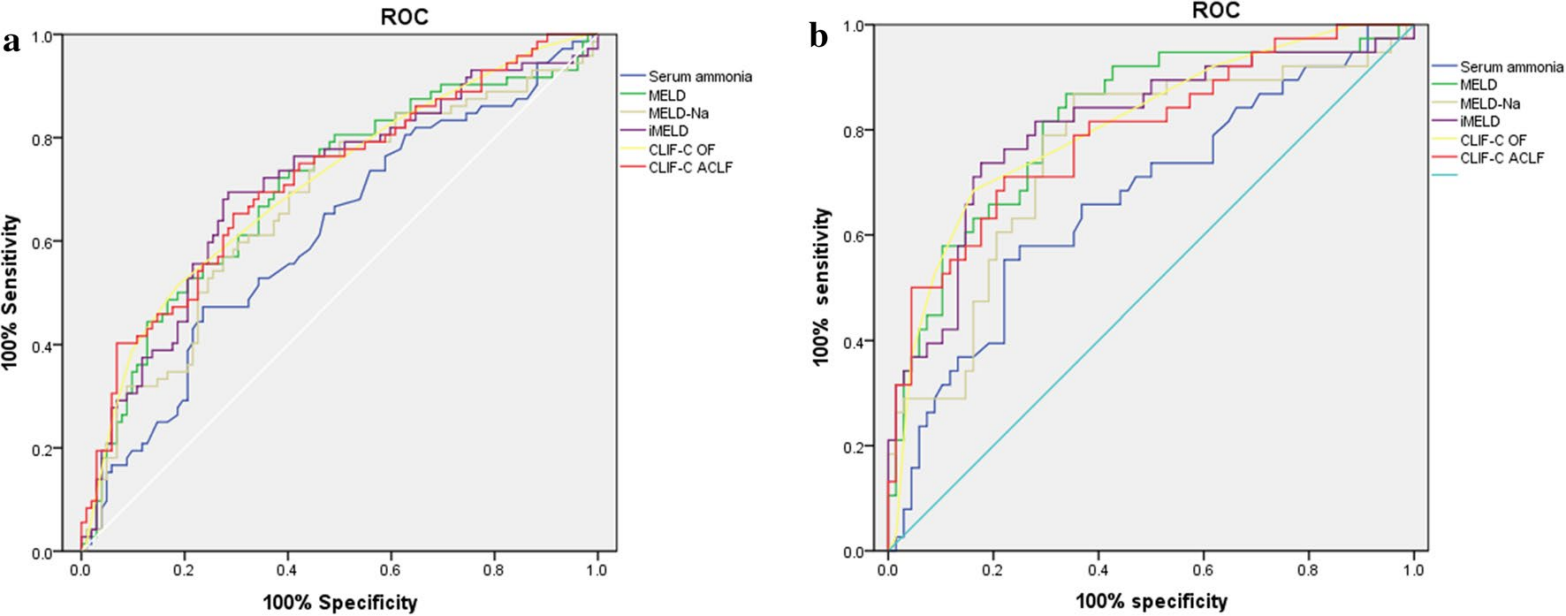
ROC curves of prognostic models in predicting 28-day mortality in ACLF groups (**a**) and HBV reactivation-induced ACLF groups (**b**).

The predictive value of serum ammonia level and other prognostic scores were examined by ROC curve analysis and comparisons. The area under the curve (AUC) for ammonia alone was 0.614 (0.528–0.699), which was comparable to the MELD-Na score (0.669 (0.586–0.752)), MELD score (0.701 (0.620–0.782)), iMELD score (0.707 (0.627–0.787)), CLIF-C OF score (0.709 (0.631–0.788)) and CLIF-C ACLF score (0.715 (0.637–0.793)) (Fig. 3a). The predictive value of serum ammonia level and other prognostic scores were examined by ROC curve analysis and comparisons. The AUC for ammonia alone was 0.669 (0.56–0.778), which was comparable to the MELD-Na score (0.755 (0.655–0.856)), MELD score (0.816 (0.729–0.903)), iMELD score (0.806 (0.714–0.898)), CLIF-C OF score (0.807 (0.719–0.895)) and CLIF-C ACLF score (0.793 (0.702–0.885)) (Fig. 3b).

### Patients' baseline characteristics for HBV reactivation-induced ACLF

Baseline demographic characteristics are shown in Table 4. Overall mortality at 28 days was 35.8% (n = 38). Nonsurvivors were older (54.58 ± 11.85 versus 49.71 ± 10.05, P = 0.027). Nonsurvivors had a higher frequency of organ failure (liver, brain, coagulation; P < 0.01). The incidence of HE was higher in nonsurvivors than survivors (73.7% versus 26.5%, P < 0.001). Ammonia, white blood cell (WBC), neutrophil, TB, DB, lactic dehydrogenase (LDH), hydroxybutyrate dehydrogenase (HBDH), international normalized ratio (INR), PT, and D-dimer levels were higher in nonsurvivors than in survivors (P < 0.05). TSH, lymphocytes, albumin, triglycerides and VLDL were lower in nonsurvivors than in survivors (P < 0.05). All prognostic scores, including MELD, MELD-Na, iMELD, CLIF-OF and CLIF-C ACLF scores, were higher in nonsurvivors than in survivors (P ≤ 0.001).

**Table 4:** Comparison of demographic and clinical characteristics of patients at diagnosis of ACLF induced by HBV reactivation.

Baseline characteristics	Survivors (N = 68)	Nonsurvivors (N = 38)	P
**Predisposition**			
Age	49.71 ± 10.05	54.58 ± 11.85	0.027
Male sex	51 (75%)	28 (73.7%)	0.881
**Complications, N (%)**			
SBP	12 (17.6%)	11 28.9%)	0.176
Gastrointestinal hemorrhage	5 (7.4%)	2 (5.3%)	1
Ascites	66 (97.1%)	38 (100%)	0.536
Infection	4 (5.9%)	5 (13.2%)	0.355
HE	18 (26.5%)	28 (73.7%)	< 0.001
**Organ failure, N (%)**			
Liver	49 (72.1%)	36 (94.7%)	0.005
Kidney	0 (0%)	1 (2.6%)	0.358
Cerebral	4 (5.9%)	17 (44.4%)	< 0.001
Coagulation	11 (16.2%)	18 (47.4%)	0.001
Circulation	4 (5.9%)	5 (13.2%)	0.355
Lung	6 (8.8%)	1 (2.6%)	0.417
**Laboratory parameters**			
Serum ammonia (μmol/L)	69.76 ± 42.78	96.05 ± 46.48	0.004
TSH (mIU/L)	0.91 ± 0.69	0.53 ± 0.65	0.006
WBC (10e9/L)	6.76 ± 3.70	8.18 ± 3.21	0.049
Neutrophil (10e9/L)	4.80 ± 3.35	6.43 ± 3.12	0.015
Lymphocyte (10e9/L)	1.24 ± 0.60	0.96 ± 0.48	0.013
Albumin (g/L)	31.48 ± 4.89	29.60 ± 3.48	0.024
TB (mg/dL)	17.68 ± 7.64	23.80 ± 6.82	< 0.001
DB (mg/dL)	12.59 ± 5.64	17.18 ± 4.96	< 0.001
TG (mmol/L)	1.36 ± 0.56	1.14 ± 0.37	0.029
VLDL (mmol/L)	1.10 ± 0.61	0.86 ± 0.44	0.027
LDH (U/L)	237.31 ± 93.67	284.53 ± 89.27	0.013
HBDH (U/L)	192.13 ± 76.20	228.61 ± 71.50	0.017
INR	2.03 ± 0.42	2.62 ± 0.86	< 0.001
PT (s)	22.86 ± 4.53	29.60 ± 9.28	< 0.001
D-dimer (µg/L)	2792.06 ± 2773.08	5674.29 ± 8310.25	0.044
**Prognostic score**			
MELD	21.38 ± 4.37	28.07 ± 7.11	< 0.001
MELD-Na	17.54 ± 7.22	25.81 ± 10.23	< 0.001
iMELD	40.10 ± 5.67	48.95 ± 10.06	< 0.001
CLIF-C OF	8.15 ± 1.76	10.53 ± 2.09	< 0.001
CLIF-C ACLF	38.09 ± 7.75	49.35 ± 11.30	< 0.001

### Associations of clinical parameters and prognostic scoring systems with serum ammonia in HBV reactivation-induced ACLF patients

Overall, 37 patients (33.3%) had baseline ammonia ≥ 92.5 µmol/L (Table 5). In the group with ammonia ≥ 92.5 µmol/L, the incidence of overt HE was higher than that in patients with ammonia ≤ 92.5 µmol/L (P < 0.001). An ammonia level ≥ 92.5 µmol/L was associated with a higher frequency of organ failures (cerebral 44.4% versus 7.1% (P < 0.001); coagulation 50% versus 15.7% (P < 0.001)). In the group with ammonia ≥ 92.5 µmol/L, ferritin, aspartate aminotransferase (AST), WBC, INR, and PT levels were higher than those the group with ammonia ≤ 92.5 µmol/L (P < 0.05), while TSH, TG, and VLDL were lower than those in the group with ammonia ≤ 92.5 µmol/L (P < 0.05). The prognostic scores, including MELD, iMELD, CLIF-C OF and CLIF-C ACLF, were higher in patients with ammonia ≥ 92.5 µmol/L than in those with ammonia ≤ 92.5 µmol/L (P < 0.01).

**Table 5:** Comparison of demographic and clinical characteristics in ACLF patients induced by HBV reactivation with and without elevated ammonia.

	Ammonia < 92.5 (n = 69)	Ammonia ≥ 92.5 (n = 37)	P
Male sex	52 (74.3%)	27 (75%)	0.936
**Complications, N (%)**			
HE	20 (28.6%)	26 (72.2%)	< 0.001
**Laboratory parameters**			
Ferritin (ng/mL)	2184.18 ± 1900.79	3894.96 ± 3519.22	0.008
TSH (mIU/L)	0.88 ± 0.74	0.56 ± 0.56	0.014
WBC (10e9/L)	6.75 ± 3.33	8.24 ± 3.86	0.041
AST	220.64 ± 179.33	311.73 ± 252.35	0.034
TG (mmol/L)	1.36 ± 0.55	1.13 ± 0.40	0.024
VLDL (mmol/L)	1.12 ± 0.59	0.82 ± 0.48	0.01
INR	2.14 ± 0.73	2.43 ± 0.50	0.032
PT (s)	24.14 ± 7.95	27.40 ± 5.57	0.029
**Organ failures**			
Liver	53 (75.7%)	32 (88.9%)	0.107
Kidney	0 (0%)	1 (2.8%)	0.34
Cerebral	5 (7.1%)	16 (44.4%)	< 0.001
Coagulation	11 (15.7%)	18 (50%)	< 0.001
Circulation	3 (4.3%)	6 (16.7%)	0.072
Lung	4 (5.7%)	3 (8.3%)	0.687
**Prognostic score**			
MELD	22.48 ± 5.83	26.19 ± 6.67	0.004
MELD-Na	19.23 ± 8.30	22.88 ± 10.59	0.053
iMELD	41.68 ± 7.60	46.24 ± 9.68	0.009
CLIF-C OF	7.78 ± 1.63	10.19 ± 1.98	< 0.001
CLIF-C ACLF	37.29 ± 8.18	47.59 ± 10.33	< 0.001
28-day mortality	17 (24.3%)	21 (58.3%)	0.001

**Figure 4:**
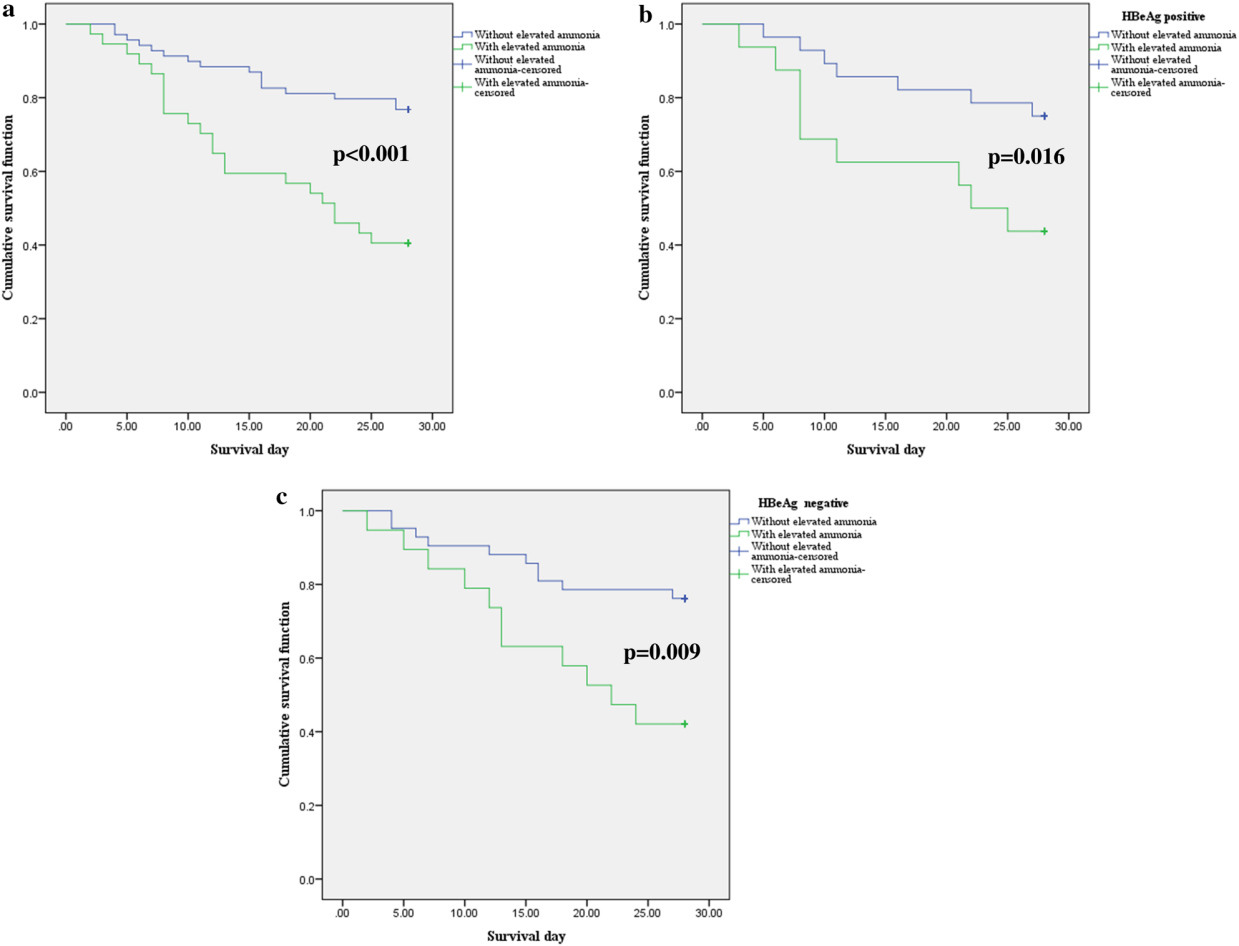
Kaplan–Meier graph of 28-day survival stratified by ammonia level. Cumulative survival across groups was compared using the log-rank test. (**a**) Twenty-eight-day survival of HBV reactivation-induced ACLF. (**b**) Twenty-eight-day survival of HBV reactivation-induced ACLF with HBeAg positivity. (**c**) Twenty-eight-day survival of HBV reactivation-induced ACLF patients who were HBeAg negative.

Patients with higher levels of ammonia had increased mortality at 28 days compared to those with normal ammonia; the phenomenon can also be observed after classification of the population into HBeAg-positive subgroup and HBeAg-negative subgroup. The survival curves stratified by ammonia level are presented in Fig. 4.

### Univariate analyses and multivariable analysis of risk factors from laboratory tests

The results showed that in univariate analyses, relatively older age (OR 1.043, P = 0.032) and higher levels of ammonia (OR 1.013, *P* = 0.006), neutrophils (OR 1.162, P = 0.024), TB (OR 1.119, P < 0.001), DB (OR 1.166, P < 0.001), creatinine (Cr) (OR 5.893, *P* = 0.009), LDH (OR 1.006, *P* = 0.017), HBDH (OR 1.007, *P* = 0.022), creatine kinase (CK) (OR 1.005, P < 0.001), INR (OR 5.377, *P* < 0.001), and PT (OR 1.178, *P* < 0.001) were high-risk factors for 28 mortality in patients with HBV reactivation-induced ACLF. On the other hand, the relatively lower levels of TSH (OR 0.381, *P* = 0.01), lymphocytes (OR 0.359, *P* = 0. 017), albumin (OR 0.904, P = 0.043), TG (OR 0.33, P = 0.035), VLDL (OR 0.445, *P* = 0.047) and D-dimer (OR 1, P = 0.04) were the high-risk factors for 28-day mortality in patients with HBV reactivation-induced ACLF. However, in multivariable Cox regression, only age (OR 1.042, P = 0.026), serum ammonia (OR 1.010, P = 0.016), lymphocytes (OR 0.396, P = 0.033), Cr (OR 4.189, P = 0.003), INR (OR 0, P = 0.003), PT (OR 2.44, P = 0.002), and D-dimer (OR 1, P = 0.02) were independent risk factors for 28 mortality in patients with HBV reactivation-induced ACLF (Table 6).

**Table 6:** Univariate and multivariate analysis of predictors of 28-day mortality.

	Unadjusted HR	P	Adjusted HR	P
Age	1.043	0.032	1.042	0.026
**Laboratory parameter**				
Serum ammonia (μmol/L)	1.013	0.006	1.010	0.016
TSH (mIU/L)	0.381	0.01		
WBC (10e9/L)	1.118	0.059		
Neutrophil (10e9/L)	1.162	0.024		
Lymphocyte (10e9/L)	0.359	0.017	0.396	0.033
Albumin	0.904	0.043		
TB (mg/dL)	1.119	< 0.001		
DB (mg/dL)	1.166	< 0.001		
Cr (mg/dL)	5.893	0.009	4.189	0.003
TG (mmol/L)	0.33	0.035		
VLDL (mmol/L)	0.445	0.047		
LDH (U/L)	1.006	0.017		
HBDH (U/L)	1.007	0.022		
CK (U/L)	1.005	0.04		
INR	5.377	< 0.001	0	0.003
PT (s)	1.178	< 0.001	2.44	0.002
D-dimer (µg/L)	1	0.04	1	0.02

## Discussion

Early in 2003, Nicolao et al. stated that there is only a rough and inconstant correlation between hyperammonemia and the clinical severity of HE^23^. EASL/AASLD clinical practice guidelines^17^ state that high blood-ammonia levels do not add any diagnostic staging or prognostic value in HE patients with chronic liver disease. They also state that HE cannot be diagnosed according to serum ammonia level since the ammonia level is in the normal range in some patients with clinical HE and neurological abnormalities. Recently, another study debated this opinion and stated that serum ammonia is related to the clinical grade of HE and serves as a prognostic factor in patients with liver cirrhosis^2^. The high mortality of ACLF patients with hyperammonemia is potentially related to the toxicity of ammonia in the circulatory system, which extends beyond the brain^24^, including a role in the pathophysiology of portal hypertension^25^. It is worth noting that the usefulness of measuring serum ammonia in routine practice remains controversial, although multiple studies highlighted that ammonia is a prognostic factor in patients with liver cirrhosis. Based on the results from our study, we measured serum ammonia in the population with ACLF, which strongly indicates their prognosis.

Hyperammonemia occurs in patients with liver cirrhosis due to reduced activity of urea cycle enzymes in the liver or portosystemic shunting^26^. Ravi et al. demonstrated that a higher level of ammonia at admission served as an important indicator of in-hospital survival in patients with alcoholic hepatitis^27^. A certain proportion of cirrhotic patients develop HE, 43% of HE patients die within 1 year, and the short-term mortality rate is extremely high in patients with more advanced grades of HE^13^. A quality meta-analysis from the Cochrane Database System Review revealed that the prevention and treatment of HE with l-ornithine l-aspartate significantly reduced the mortality of patients with cirrhosis with acute decompensation when compared to patients treated with placebo, and this treatment decreased the serum level of ammonia to improve the survival rates of these patients^28^. In addition, several studies found that the incidence of HE is closely related to the mortality of patients with chronic liver diseases^29,30^. However, they speculated that a significant correlation exists between increasing ammonia levels and the development of HE or the severity of HE. Two recent studies highlighted that ammonia levels on admission are important predictive factors of in-hospital mortality in decompensated cirrhosis^6,27^.

Currently, no study has directly analyzed the prognostic effect of ammonia in populations with APASL ACLF. Our study adds to these previous studies through inclusion of a defined ACLF population and further analysis of HBV reactivation-related ACLF, allowing for analysis of the relationship between ammonia and various prognostic factors in the population. We proved that hyperammonemia is a strong indicator in the prognosis of patients with ACLF, which indicated that lowering ammonia in these patients may prolong the survival time of ACLF regardless of the incidence of HE. Although the data were obtained retrospectively from the studies, all patients were prospectively recruited. In the total ACLF population, an ammonia level of ≥ 89 µmol/L is closely correlated with liver, coagulation, and brain failure, although our data did not find a relationship between higher ammonia and kidney, circulation or respiration failure. Although there is no difference in HE incidence between ACLF with ammonia level of ≥ 89 µmol/L and those without ammonia level of ≥ 89 µmol/L, we found that ammonia level is positively related to HE grade according to CLIF-OF scores. This is in accordance with the most recent study by Shalimar et al.^2^. HBV infection is still an important public health problem since 3/4 of people with HBV infection and chronic carriers with positive HBV surface antigens (HBsAg) are from China. On the other hand, nearly 50,000 people die because of HBV infection every year in China^31^. This reminds us that HBV reactivation is a life-threatening case in the current status. After we analyzed the data from our hospital, we found that most patients underwent progression into ACLF when they withdrew or accepted irregular usage of nucleoside analogs. In addition, HBV reactivation commonly occurs in other patients undergoing impairment of their antiviral immunity, such as chemotherapy, immunosuppressive treatment or biological therapy^32^. Without considering clinical signs of HE or other parameters in ACLF patients, a high level of ammonia indicates a significantly higher risk of death at 28 days. We found that ammonia is still a strong prognostic factor in HBV reactivation-induced ACLF and is closely related to more prognostic scores according to our study. The Kaplan–Meier survival curves further demonstrated that ammonia was still a strong prognostic factor after division of the population into subgroups.

In vivo and in vitro studies showed that ornithine phenylacetate significantly decreased the cell death rate of hepatocytes and alleviated the progression of fibrosis after downregulation of serum ammonia levels^33^. Although the pathophysiology of HE is multifactorial and undetermined according to current studies, hyperammonemia, inflammation and genetic factors are three main pathways in the pathogenetic process. In bile duct-ligated rats, hyperammonemia-induced brain edema and brain swelling can be reduced by a reduction in ammonia^34^. In another model of liver cirrhosis, downregulation of ammonia levels protected the brain from a subsequent challenge with lipopolysaccharide^35^. This indicates that ammonia is a potential goal of treatment to prolong the survival time of ACLF patients.

However, there are still limitations in this study, as we appealed to expand the study population in future study. The cut-off of the actual ammonia level should be further determined according to additional larger studies around the world. In an Indian study^36^, a cut-off value of 124 µmol/L upon initial evaluation predicted death in 76% of cases. In 2007, Bernal et al. showed that an arterial ammonia level above 100 µmol/L is the cut-off that sensitively and specifically predicts the occurrence of severe HE in 70% of cases^37^. In 2019, another study demonstrated that an arterial ammonia level above 79.5 µmol/L is the cut-off that sensitively and specifically predicts the outcome of patients with liver cirrhosis^2^.

To this end, this study of ACLF patients adds significantly to the evidence that ammonia levels correlate not only with the failure of other organs but also with 28-day mortality. A reduction in ammonia levels may serve as a potential therapeutic target in patients with ACLF.

## References

[1] Clemmesen JO, Larsen FS, Kondrup J, Hansen BA, Ott P (1999). Cerebral herniation in patients with acute liver failure is correlated with arterial ammonia concentration. Hepatology (Baltimore, MD).

[2] Shalimar (2019). Prognostic role of ammonia in patients with cirrhosis. Hepatology (Baltimore, MD).

[3] Shawcross DL (2011). Infection and systemic inflammation, not ammonia, are associated with Grade 3/4 hepatic encephalopathy, but not mortality in cirrhosis. J. Hepatol..

[4] Vierling JM (2016). Fasting blood ammonia predicts risk and frequency of hepatic encephalopathy episodes in patients with cirrhosis. Clin. Gastroenterol. Hepatol..

[5] Sawhney R (2016). Role of ammonia, inflammation, and cerebral oxygenation in brain dysfunction of acute-on-chronic liver failure patients. Liver Transpl..

[6] Patwardhan VR (2016). Serum ammonia in associated with transplant-free survival in hospitalized patients with acutely decompensated cirrhosis. J. Clin. Gastroenterol..

[7] Zhao RH, Shi Y, Zhao H, Wu W, Sheng JF (2018). Acute-on-chronic liver failure in chronic hepatitis B: An update. Expert Rev. Gastroenterol. Hepatol..

[8] Sarin SK (2019). Acute-on-chronic liver failure: Consensus recommendations of the Asian Pacific association for the study of the liver (APASL): An update. Hepatol. Int..

[9] Shi Y (2015). Acute-on-chronic liver failure precipitated by hepatic injury is distinct from that precipitated by extrahepatic insults. Hepatology (Baltimore, MD).

[10] Li H (2016). Characteristics, diagnosis and prognosis of acute-on-chronic liver failure in cirrhosis associated to hepatitis B. Sci. Rep..

[11] Hwang JP, Lok AS (2014). Management of patients with hepatitis B who require immunosuppressive therapy. Nat. Rev. Gastroenterol. Hepatol..

[12] Kumar M, Jain S, Sharma BC, Sarin SK (2006). Differentiating acute hepatitis B from the first episode of symptomatic exacerbation of chronic hepatitis B. Dig. Dis. Sci..

[13] Moreau R (2013). Acute-on-chronic liver failure is a distinct syndrome that develops in patients with acute decompensation of cirrhosis. Gastroenterology.

[14] Blei AT, Cordoba J (2001). Hepatic encephalopathy. Am. J. Gastroenterol..

[15] Nahon P (2017). Bacterial infection in compensated viral cirrhosis impairs 5-year survival (ANRS CO12 CirVir prospective cohort). Gut.

[16] Ong JP (2003). Correlation between ammonia levels and the severity of hepatic encephalopathy. Am. J. Med..

[17] Vilstrup H (2014). Hepatic encephalopathy in chronic liver disease: 2014 practice guideline by the american association for the study of liver diseases and the European Association for the Study of the liver. Hepatology (Baltimore, MD).

[18] Polson J, Lee WM (2005). AASLD position paper: The management of acute liver failure. Hepatology (Baltimore, MD).

[19] Wiesner R (2003). Model for end-stage liver disease (MELD) and allocation of donor livers. Gastroenterology.

[20] Biggins SW (2006). Evidence-based incorporation of serum sodium concentration into MELD. Gastroenterology.

[21] Luca A (2007). An integrated MELD model including serum sodium and age improves the prediction of early mortality in patients with cirrhosis. Liver Transpl..

[22] Jalan R (2014). Development and validation of a prognostic score to predict mortality in patients with acute-on-chronic liver failure. J. Hepatol..

[23] Nicolao F (2003). Role of determination of partial pressure of ammonia in cirrhotic patients with and without hepatic encephalopathy. J. Hepatol..

[24] Dasarathy S (2017). Ammonia toxicity: From head to toe?. Metab. Brain Dis..

[25] Jalan R (2016). Ammonia produces pathological changes in human hepatic stellate cells and is a target for therapy of portal hypertension. J. Hepatol..

[26] Weiss N, Jalan R, Thabut D (2018). Understanding hepatic encephalopathy. Intens. Care Med..

[27] Ravi S, Bade KS, Hasanin M, Singal AK (2017). Ammonia level at admission predicts in-hospital mortality for patients with alcoholic hepatitis. Gastroenterol. Rep..

[28] Goh ET (2018). L-ornithine L-aspartate for prevention and treatment of hepatic encephalopathy in people with cirrhosis. Cochrane Database Syst. Rev..

[29] Cordoba J (2014). Characteristics, risk factors, and mortality of cirrhotic patients hospitalized for hepatic encephalopathy with and without acute-on-chronic liver failure (ACLF). J. Hepatol..

[30] Romero-Gomez M, Montagnese S, Jalan R (2015). Hepatic encephalopathy in patients with acute decompensation of cirrhosis and acute-on-chronic liver failure. J. Hepatol..

[31] Arzumanyan A, Reis HM, Feitelson MA (2013). Pathogenic mechanisms in HBV- and HCV-associated hepatocellular carcinoma. Nat. Rev. Cancer.

[32] Pawlowska M (2019). Prophylaxis of hepatitis B virus (HBV) infection reactivation—Recommendations of the Working Group for prevention of HBV reactivation. Clin. Exp. Hepatol..

[33] De Chiara F (2019). Ammonia scavenging prevents progression of fibrosis in experimental nonalcoholic fatty liver disease. Hepatology (Baltimore, MD).

[34] Bosoi CR, Parent-Robitaille C, Anderson K, Tremblay M, Rose CF (2011). AST-120 (spherical carbon adsorbent) lowers ammonia levels and attenuates brain edema in bile duct-ligated rats. Hepatology (Baltimore, MD).

[35] Wright G (2012). Reduction in hyperammonaemia by ornithine phenylacetate prevents lipopolysaccharide-induced brain edema and coma in cirrhotic rats. Liver Int..

[36] Bhatia V, Singh R, Acharya SK (2006). Predictive value of arterial ammonia for complications and outcome in acute liver failure. Gut.

[37] Bernal W (2007). Arterial ammonia and clinical risk factors for encephalopathy and intracranial hypertension in acute liver failure. Hepatology (Baltimore, MD).

